# Feasibility, Acceptability and Clinical Utility of the Bereavement and Grief Cultural Formulation Interview for Prolonged Grief Disorder

**DOI:** 10.1007/s11013-025-09927-2

**Published:** 2025-07-28

**Authors:** Clare Killikelly, Lea-Martina Christen, Simon Groen, John S. Ogrodniczuk, Andreas Maercker, Geert E. Smid, Eva Heim

**Affiliations:** 1 Department of Psychology, University of Zurich, Binzmuehlestrasse 14/17, CH-8050 Zurich, Switzerland; 2 Department of Psychiatry, University of British Columbia, Vancouver, Canada; 3 De Evenaar, Center for Transcultural Psychiatry, GGZ Drenthe, Assen, Netherlands; 4 ARQ National Psychotrauma Centre, Diemen, The Netherlands; 5 University of Humanistic Studies, Utrecht, The Netherlands; 6 Institut de Psychologie, Université de Lausanne, Lausanne, Switzerland

**Keywords:** Prolonged grief disorder, Cultural formulation interview, Feasiblity, Clinical interview, Assessment, Diagnosis, Culture

## Abstract

Prolonged grief disorder (PGD) is a new diagnostic category included in global diagnostic classification systems for mental disorders. However, PGD can only be diagnosed if the severity and duration exceed socio-cultural norms. Here, we present a new supplementary module to the DSM-5 Cultural Formulation Interview: the Bereavement and Grief Cultural Formulation Interview (BG-CFI). The BG-CFI was developed to help clinicians provide a culturally informed diagnosis and guide treatment planning.

We investigated the feasibility, acceptability, and clinical utility of the BG-CFI. Two participant groups (11 refugees, asylum seekers or migrants experiencing bereavement and 3 clinicians) took part in the study and were interviewed using open-ended questions on measures of feasibility, acceptability, and clinical utility. A step-by-step procedure was followed: (1) Clinicians and/or researchers conducted the BG-CFI with participants; (2) Debriefing interviews were conducted separately with clinicians and with bereaved participants.

The BG-CFI was found to be a feasible, acceptable, and clinically useful tool for both bereaved participants and clinicians. Where clinicians found the interview difficult to conduct (i.e. lack of conceptual clarity or triggering emotional distress) specific changes were made to the interview format such as prompts for further questioning or recommendations for withholding or adapting questions. The BG-CFI would offer a useful complement for a reliable assessment of PGD in clinical settings working with cultural incongruity.

**Supplementary Information:**

The online version contains supplementary material available at 10.1007/s11013-025-09927-2.

## Introduction

Prolonged grief disorder (PGD) is a newly recognized mental health disorder recently included in worldwide diagnostic classification systems. Both the 11th edition of the International Classification of Diseases (ICD-11) from the World Health Organization and the latest revision of the Diagnostic and Statistical Manual of Mental Disorders (American Psychiatric Association, 2022; World Health Organization, 2020) have defined PGD in terms of similar core and accessory symptoms, time, and impairment criteria and the cultural caveat (Killikelly & Maercker, 2017). Building on the need for increased global applicability of mental health diagnosis, the cultural caveat states that PGD can only be diagnosed if the symptom presentation is beyond the expected to duration and intensity defined by the socio-cultural norms of the individual. More precisely the ICD-11 states *‘The grief response has persisted for an atypically long period of time following the loss (more than 6 months at a minimum) and clearly exceeds expected social, cultural or religious norms for the individual’s culture and context*’(World Health Organization, 2020). Similarly, the DSM-5-TR states that PGD can only be diagnosed when ‘*markedly exceeding expected social, cultural or religious norms for the individual’s culture and context. Grief responses lasting for less than 6 months, and for longer periods in some cultural contexts, should not be regarded as meeting this requirement*’ (APA *DSM*-5-TR: the *Diagnostic and Statistical Manual of Mental Disorders* 5, Text Revision)

The cultural caveat acknowledges the fact that (sociocultural) context considerably shapes the experience and expression of mental distress. Although PGD is the first diagnostic category for which such a cultural caveat of the ICD-11 was introduced, there is growing consensus that contextual information is most relevant for diagnosis and treatment of mental disorders. In this spirit, DSM-5 introduced the term “cultural concepts of distress” (CCD). CCD are defined as contextualized ways of experiencing, expressing, and understanding psychological distress, which includes idioms of distress, explanatory models, and beliefs about help seeking (Kohrt et al., 2014; Lewis-Fernández & Aggarwal, 2013; Rasmussen et al., 2014).

Lewis-Fernández and Kirmayer (2019) define the relationship between diagnostic categories of mental disorders (as described in ICD and DSM) and CCD in a sense that the former are abstract and decontextualized (i.e. nomothetic) constructs, whereas CCD are contextualized (idiographic) presentations. As an example, the (relatively abstract) diagnostic criteria for complex post-traumatic stress disorder (CPTSD) as defined in ICD-11 can be contextualized in terms of different presentations across different settings (Bovey et al., 2024; Hosny et al., 2023).

In a similar manner, there is large cultural variation in symptoms of grief (Rosenblatt, 2008) and several research groups have identified CCD related to grief and bereavement, including culturally specific symptoms (Bolton, 2001; Tsutsui et al., 2014; Wikan, 1988; Xiu et al., 2016). Our research has found that different cultural groups endorse different symptoms of grief (Killikelly & Maercker, 2023). For example, a network analysis found that a sample of bereaved adults in China strongly endorsed somatic symptoms (i.e. headache and fatigue) (Stelzer et al., 2020a, 2020b), whereas a sample of bereaved refugees from Syria reported that ‘emotional outbursts’ related to anger and blame were common (Killikelly et al., 2021).

Importantly, what is deemed normal in one cultural context may be deemed abnormal and therefore as an indicator of mental illness in another (Groen et al., 2022). For example, our key informant interviews with a sample of bereaved individuals from Japan revealed that yearning for a loved one who had passed away was a core phenomenological experience of grief. However, health care workers in Japan expressed concern about yearning as a core diagnostic symptom of ICD-11 PGD pathology. Expressive forms of yearning, such as ‘butsudan’ (ritual practice involving alter worshiping for the deceased) is a very normal and healthy practice for individuals living in Japanese society and may be practiced for years after the death of a loved one (Killikelly et al., 2022). In this context, intense yearning for deceased, as defined by the DSM-5 as a core symptom of PGD, would not necessarily be considered problematic.

As a reference point for clinicians, the DSM-5 included several possible CCD in a catalogue-like list for clinicians (Aggarwal, 2013). This is a useful starting point, but the influence of cultural factors extends beyond the individual symptom level to the system of mental health assessment. The process of mental health assessment (i.e. visiting a clinic, speaking with a health care professional, completing a questionnaire) is part of a cultural practice that may be unfamiliar to some socio-cultural groups. Several researchers and clinicians have critiqued the concept of culture associated with CCD, as it places a strong emphasis on the individual participant while neglecting the wider structural and systemic influence of the culture of modern psychiatry (Aggarwal, 2013).

Across our research studies, we have consistently found a certain level of acquiescence whereby individuals may believe they honor their deceased loved one by indicating strong and persistent levels of grief on standard questionnaires (Killikelly et al., 2020; Stelzer et al., 2020a, 2020b). To demonstrate recovery or indicate low levels of grief on a questionnaire may seem like ‘moving on’ or accepting the loss. For example, for individuals living in a Chinese cultural context, filial piety would prescribe intense mourning and display of grief publicly and privately to demonstrate honor and obedience to one’s ancestors (Stelzer et al., 2020a, 2020b). This highlights the importance of considering cultural norms and expectations in clinical encounters. The expression and experience of distress may depend on cultural context, but it will also depend on the individual’s interaction with a clinician and the clinical setting. For example, the use of specific idioms of distress may serve a particular function. Zhou et al., (2016), explored the endorsement of somatic symptoms (e.g. headaches, fatigue) in a sample of individuals from China with depression. They discovered that the expression of somatic distress did not necessarily indicate physical pain but was considered to be a more culturally acceptable way to express distress. Ultimately this led to quicker access to health care support.

It is increasingly understood that culture influences every aspect of the clinical encounter (Cooke, 2018; Kirmayer & Ban, 2013; Lewis-Fernández et al., 2017). A recent model – the cultural ecosystems model of psychiatry – seeks to explore the relationship between the individual and several levels of environmental factors (Gómez-Carrillo & Kirmayer, 2023). This model moves away from the traditional neurobiological reductionist view of mental disorders toward the inclusion of social, behavioral, and systemic determinants. The authors propose a hierarchical ordering of factors that may influence the experience and expression of mental disorder symptoms at any level. For example, they include processes such as an individual’s narrative self-construal and responses to socio-cultural factors such as family system, neighborhoods, and health care system as factors that may influence symptom presentation.

Against this background, the question of how to reliably operationalize the cultural caveat in every day clinical practice remains challenging. The DSM-IV Outline for Cultural Formulation (OCF) and the DSM-5 Cultural Formulation Interview (CFI) represent notable efforts developed in close collaboration by scholars and clinicians from the fields of psychiatry, psychology, and anthropology to more reliably assess and evaluate psychological distress and mental disorders across different cultural groups (Lewis-Fernández, 1996; Lewis-Fernández et al., 2017). These clinical interview guidelines aim at establishing a shared model of psychopathology that is co-created through the interaction between the participant and clinician (Aggarwal, 2023). A recent scoping review of the use of the CFI since publication in 2013 found that 30 studies have confirmed its usefulness in establishing rapport between participants and clinicians, exploring patients’ illness narratives and improving treatment planning (Jones-Lavallee et al., 2023). A series of Supplementary Modules to the CFI were developed, e.g. modules exploring ‘the explanatory model’, level of functioning, social network, cultural identity, modules relevant to older adults and a module for migrants and refugees (Lewis-Fernández et al., 2015).

To address a key clinical gap in assessing Prolonged Grief Disorder (PGD) across cultures, a Supplementary Module to the Cultural Formulation Interview (CFI) was developed in 2018: the *Bereavement and Grief – Cultural Formulation Interview* (BG-CFI) (Groen et al., 2022; Smid et al., 2018a, 2018b). The BG-CFI was designed to facilitate an in-depth, culturally sensitive assessment of bereavement and grief in individuals who may experience PGD.

The initial draft was developed by psychiatrist Prof. Geert Smid and anthropologist Dr. Simon Groen—who also contributed to the original CFI (Lewis-Fernández et al., 2017)—in collaboration with experts in cultural psychiatry (Simon M. de la Rie) and PGD (Paul Boelen). The development was guided by DSM-5 criteria for Persistent Complex Bereavement Disorder (PCBD) and its culture-related diagnostic issues section.

The BG-CFI incorporated insights from clinical experiences with culturally diverse bereaved individuals, including differences between cultural norms of the country of origin and the host culture (culture incongruity). These informed both the content and formulation of questions.

The draft has undergone expert revisions and iterative testing in current and previous studies (Groen et al., 2022). It is intended as a semi-structured interview to:

1. Help clinicians assess the role of grief in mental health,
2. Explore the significance of rituals or their absence,
3. Understand expectations of support and help-seeking behavior,
4. Foster shared understanding and decision-making around grief interventions, and
5. Improve rapport and therapeutic outcomes (Smid et al., 2018a, 2018b).

The aim of the current study is to pilot the draft BG-CFI in a group of clinicians and bereaved participants (refugees, migrants or asylum seekers experiencing bereavement) to examine the feasibility, acceptability, and clinical utility of the BG-CFI to establish a more reliable assessment of PGD in various cultural contexts.

## Methods

### Procedure

A qualitative approach was chosen to examine the feasibility, acceptability, and clinical utility of the semi-structured, in-depth Bereavement and Grief Cultural Formulation Interview (BG-CFI) by Smid et al., (2018a, 2018b). Semi-structured interviews provide a narrative approach that can afford a more complete understanding of the individuals unique experience. In addition, due to the sensitive nature of the topic (grief, migration, loss) interviews were deemed to be more appropriate than self-report questionnaires. Two participant groups (clinician-participants and refugees, asylum seekers or migrants experiencing bereavement for brevity termed bereaved participants throughout the paper) took part in the study and were interviewed using open-ended questions on measures of feasibility, acceptability, and clinical utility. A step-by-step procedure was followed: (1) clinicians and/or researchers conducted the BG-CFI with participants; (2) Debriefing interviews were conducted by lead researcher LC individually with clinicians, called “Debriefing Interview for Clinicians” (DIC), and bereaved people, called “Debriefing Interview for Participant/Patient” (DIP). These debriefing interviews comprised 11 questions based on original DIP/DIC from Lewis-Fernandez et al., 2017 however slightly adapted to the BF-CFI, i.e. Question 8. Are there any particular BG-CFI questions that you think should be changed or removed, perhaps because they were unclear?

In the current study participants only completed the BG-CFI as the full Cultural Formulation Interview of the DSM is lengthy and not part of the main research question. The debriefings were all conducted by researchers of the study team. All but one interview were conducted either online via the video call program Zoom or via phone call. All participants and research team members were located in Switzerland. Informal interpreters were consulted when needed and interpreted live during the interview.

All participants had to sign the consent form provided by the researcher prior to participating in the study. Ethical approval was granted by the University of Zurich with the number Req-2019-00801.

### Recruitment

Clinician-participants were recruited via snowball technique and were selected through clinics and hospitals that offered psychotherapy to refugees, asylum seekers, or migrants within Switzerland. For bereaved individuals participant recruitment, refugee contact points such as asylum centers, non-governmental organizations, hospitals, and therapeutic facilities were contacted via email or phone. In addition, flyers with study information (i.e. the inclusion criteria listed below) were distributed in the greater area of Zurich. We assessed two different participant samples: One participant sample was recruited through the clinician-participants (clinical stream), the other sample included bereaved participants who were recruited by the researcher team (community stream).

In the clinical stream, clinicians conducted the BG-CFI with the bereaved participants. Afterward, the researcher conducted the debriefing separately with the bereaved participants and the clinician-participant. In the community stream, the researcher first conducted the BG-CFI and second the debriefing interview with the bereaved participants. Participants from the community stream (but not from the clinical stream) received 30 Swiss francs (CHF) for participating in the study.

#### Inclusion Criteria for Bereaved Participants

Participants were refugees, asylum seekers, or migrants, age of 18 years or older, that had experienced the death of a loved one within the past ten years. For participants recruited from the clinical stream, this death had to have occurred six months ago or longer. Participants could be included if interpreter-assistance was available.

#### Inclusion Criteria and Training for Clinician-Participants

Clinician-participants had to be fluent in German and/or English, have a qualification for working in mental health care (psychologist, psychiatrist, social worker), and had to have a minimum of one year of experience working with grief and with refugees, asylum seekers and/or migrants. After agreeing to participate in the study, clinicians went through an online training guide including vignettes on how to use the BG-CFI. A larger training package has subsequently been developed (see Groen et al., 2022). It was based on an unpublished written document (personal communication) by Prof. Geert E. Smid (2018), the first author of the BG-CFI.

### Data Collection

Data collection occurred from March to June 2020. Due to the COVID-19 pandemic contact was restricted via Zoom online interviews instead of face-to-face. The audio material was then transcribed using the program MAXQDA(VERBI, 2020). Transcripts were translated to English by researchers. Demographic data of clinicians and participants was collected via demographic data forms. The mean duration of the total collection (demographic data, BG-CFI and debriefing) per participant was 50 min. The average time needed to conduct only the BG-CFI was 27 min. The average time for the debriefings was 14 minutes for participants (DIP) and 24 min for clinicians (DIC).

### Thematic Analysis

For data analysis, the thematic analysis approach was used. This is predominantly used in psychology and the social sciences to document and define recurring themes, patterns, concepts or ideas in qualitative data. (Howitt, 2013). MAXQDA (VERBI, 2020) was used for the analysis. We applied a data-led inductive approach, since we wanted to be open to new subjects and topics, rather than directly applying a deductive theory. However, our thematic analysis was guided by the aims of assessing feasibility, acceptability, and clinical utility. We applied the six steps from the model of Braun and Clarke (2006): familiarization of data; generation of codes: combining codes into themes; reviewing themes; determining significance of themes; and reporting of the findings. Codes were generated after rereading the transcripts line by line and then grouped into themes which represent a second level of interpretation of the text. In some cases, sub-themes were generated to better explain the different aspects of a main theme. After reviewing and reorganizing all themes, they were finally defined and labeled. To establish a high intercoder-reliability, along with the first coder (LC), a second coder (IG) was nominated to simultaneously code all the data for the analysis. After each step, the codes, themes, and sub-themes were compared and discussed until an agreement was reached. In case of disagreement, the opinion of a third party was obtained (CK).

## Results

### Participants

Of the total of fourteen participants, eleven were displaced people (i.e. individuals with migrant, refugee or asylum seeker status) that had experienced the loss of a loved one, and three were clinicians.

#### Clinicians

Two of the three clinicians were female. On average, they had been working with displaced people for about 10.6 years (SD = 10.14). The average years of clinical practice experience was 14.5 years (SD = 12.11). Two clinicians conducted the interview in German and one in English. None of the clinicians had the same cultural background as their participant. All the clinicians were psychologists or psychiatrists working in a specialized mental health clinic for displaced people in Switzerland**.** Each clinician conducted two BG-CFI assessments with two different patients.

#### Participants

All of the eleven participants were displaced people with a loss experience, with or without a diagnosed PGD, living in Switzerland or Germany. Six were current patients of one of the participating clinicians, the remaining five were currently not in any therapeutic treatment. Fifty-five percent (*n* = 6) of participant-participants were female and the age ranged from 23 to 61 years with an average mean age of 38 years. Four participants were from Syria, four from Afghanistan, one from Eritrea, one from Sri Lanka, and one from Uganda. Two participants underwent the interview in English, five in German and four needed a translator. Four out of eleven participants had been able to attend the funeral after their loved one had died, and for most of them (*n* = 8), the death of their loved one was unexpected.

The results of the qualitative thematic analysis are grouped into the main categories of feasibility, acceptability, and clinical utility. Within each of these main categories, the responses from participants and clinicians are presented separately with emerging themes and sub-themes underneath. Feasibility is colloquially defined as ‘can it be done in clinical settings?’, acceptability as ‘do participants and clinicians like it?’, and clinical utility as ‘is it helpful?’ (Lewis-Fernández et al., 2017).

### Feasibility

#### Participants

##### Comfort-Distress

The first theme describes emotional states that some participants experienced during the interview. Most participants said it made them sad to talk about the deceased. In some cases, it even caused severe distress; participants sometimes started crying and the interview had to be interrupted. Others said it was a mix between sadness and happiness.

Nevertheless, seven participants said they felt comfortable overall, and no participant expressed a continuous discomfort during the interview.

“[…] but be reminded again and again, that also makes me ... that also makes me sad.” (participant 6)

“It can be both because the negative is that you think of the deceased person and the positive is how you can let go of that. And yes...” (participant 4)

##### Ease-Difficulty

Most participants found answering the questions easy, except for two participants (participants 8 and 9) who suggested adding an additional question at the end to focus on the positive experiences to conclude the interview.

“[…] in general, everything was clear and all the questions were somehow understandable.” (participant 2)

Participant 9 said he would add a question about how one has developed since the death of a loved one, talking about positive things that have happened since then. Participant 8 suggested she would add a question about how a person can deal with the distress and how one can get better.

##### Ability to Express Oneself

Five participants said the interview helped them express their emotions and/or thoughts. Three out of these five patients said that talking about the death of the loved one improved their wellbeing: “Yeah, everything was helpful, cause like, for me if I talk about myself, I share my story, I talk to someone, someone answers me, someone encourages me, I at least feel a little difference, I don’t remain that sense… towards good hope, yeah.” (participant 11)

Two patients reported having mixed feelings afterward: “On one hand it’s sadness but on the other hand it was also like little moments of happiness because of these memories which reminded you on how nice it was” (participant 2). None of the patients reported feeling worse after the interview than before. For one participant, it was the first time talking about the topic of bereavement and grief, therefore he had trouble to express his emotions: “So that was the first time for me, I can’t express my feelings, for example.” (participant 9)

##### Duration of Interview

All participants found the length of the interview to be acceptable; however, one participant said that the interview was too long (participant 9). This was also the participant who revealed this was the first time talking about this topic.

#### Clinicians

##### Comfort-Distress

This theme describes the clinicians’ evaluations of participants’ distress. Two clinicians reported a certain level of distress and vulnerability in their participants. Both had to interrupt the interview or leave out sub-questions because their participant became too distressed to continue. One clinician said that although the questions did not distress the participant too much, they recommended to only do the interview with participants that have been stabilized before to avoid re-traumatization.

This theme also describes the extent to which clinicians felt comfortable or uncomfortable addressing culture-specific aspects. All clinicians felt comfortable: “It was helpful because I knew exactly what to ask. As mentioned before, sometimes you have ambiguities in your head, but you do not know how to address the topic and here, the questions are a good help.” (Clinician 1)

##### Ease-Difficulty

This theme describes the extent to which clinicians found it easy or difficult to conduct the interview with participants. Clinicians reported several difficulties: it was sometimes difficult for clinicians to distinguish if a question addressed the participant directly or their culture. In the case of ambiguous loss, i.e. when the loved one had disappeared (and the participant had not accepted the possibility of this person being dead), some of the interview questions did not seem to be adequate. Clinician 1 struggled with question 7 (i.e. 7. What do your family, friends, and others in your community think happens after death?) because it was too distressing for the participant to continue with the sub-questions. Clinician 3 struggled to address culture-specific aspects because of language barriers or a different cultural perspective of a problem.

Clinician 1 reported “Difficult for me was, I remember, on the one hand I found it difficult in the interview to find out when is the patient meant and when is the culture meant, meaning the cultural way of dealing with things.”

Clinician 3 reported “[…] sometimes [the bereaved] do not understand the meaning or the procedure, that is to say purely linguistically [translation difficulties] and thus characterized by the European image [perhaps meaning that the clinical assessment procedure is part of Western European culture]. Sometimes that’s difficult too, I’ve felt this difficulty in many moments. And I have the impression that ... yes, these are two different worlds that meet now.”

##### Language/Cultural Barriers

This theme describes language or cultural barriers that occurred during the interview. There were several translation issues. Translators either didn’t understand the questions in the original language, or some expressions and considerations didn’t exist in the translated language. Clinician 2 criticized the interview in general to be too complicated to conduct with patients that do not speak German as the mother tongue.

##### Duration of Interview

The last theme covered clinicians’ opinions of the duration of the interview. Two clinicians found the duration acceptable, and one suggested to shorten the interview, as it was perceived to be repetitive.

Clinician 2: “(Laughs). Depends, I think, a lot on the client. Or on the patient... Well... You could also shorten it a little bit, because a lot of things came up twice.”

### Acceptability

#### Participants

##### Appropriateness/Cultural Sensitivity

Except for participant 2, who did not make a statement on this topic, all participants found the interview and its questions to be culturally appropriate.

Participant 7 said: “Well, everything was fine. All questions were good. There were no bad ones. No. No.”

##### Clarity of Questions

This theme describes clarity of the interview questions and possible ambiguities. Six participants found the questions to be clear: “[…] in general everything was clear and all the questions were somehow understandable” (Participant 2). One participant needed further explanation from her clinician (participant 8).

##### Changed/Added Questions

This theme suggests changes or additions to the interview questions. Two participants (participant 8 and 9 mentioned above) answered that they would add a question about personal growth since the death of a loved one, or how a person deals with grief.

#### Clinicians

##### Clarity of Questions

This theme describes the clarity of interview questions for clinicians and their ambiguities. Two clinicians reported that it was not clear to them if the question addressed the participant personally or their cultural background*: “I think that is not so well delimited, so if you ask how you handle it, you could understand it as how you handle it in your community, for example.”* (Clinician 1)

According to one clinician, question 4 (i.e. 4. How do your family, friends, and others in your community mourn or express their grief after the funeral/farewell?) and its sub-questions should be more specific. Another clinician reported the meaning of questions 7 to 11 were partly incomprehensible and required further content clarity.

##### Appropriateness

This theme included suggestions to add, change, or remove any questions due to cultural insensitivity. No suggestions were made, and clinicians agreed that the interview was sensitive for cultural differences.

##### Comprehensiveness

The last theme describes the comprehensiveness of the interview. Clinicians were asked if they would add a question that might be important and that was not asked in the BG-CFI. Two clinicians found that the interview was very comprehensive and addressed many important aspects. One said that a question asking whether a participant has had a grieving process at all so far is missing.

### Clinical Utility

#### Participant

##### Perception of Mental Health Care

This theme covers the extent to which the interview changed the patients’ view of mental health care. Seven participants said there was no effect on their perception. One patient said it had an effect, but without specifying in which direction.

##### Support through Clinicians

This theme describes the extent to which participants felt supported through their clinicians or researchers. Five participants reported having felt supported, the rest did not give a statement on this topic.

#### Clinicians

##### Use in Clinical Practice

Clinicians were asked directly if they would recommend including the BG-CFI in clinical practice. All the clinicians would recommend including it, depending on the circumstances of the diagnosis. Clinician 3 specified that it would be served better as an explorative instrument in a suspected PGD, rather than as a diagnostic tool. Clinician 2 said she would use it as a tool for differential diagnostics with PTSD and PGD.

##### Influence on Diagnosis and Treatment Planning

For clinician 1, conducting the BG-CFI confirmed her assumption of PGD in a participant. She replaced the original diagnosis of depression with PGD after the interview. For her, conducting the interview also confirmed her treatment plan. Another clinician said that the interview helped her acknowledging grief as a main problem:

“I would have estimated the percentage of grief rather lower, before. It is much higher than I thought.” (Clinician 2)

##### Better Understanding of Patient’s Grief

This theme describes the extent to which the interview helped the clinicians to better understand their patients’ grief. For all clinicians the BG-CFI provided valuable new information about helpful farewell rituals, patients’ mechanisms for coping with their grief and information about their culture.

##### Influence on Therapeutic Relationship

Clinician 2 reported the interview improved the therapeutic relationship. In the other cases, the effect was not clear at the time of the debriefing. Clinicians said that effects might only appear later in the therapeutic process.

## Discussion

Overall, this pilot study has found support for the feasibility, acceptability, and clinical utility of the BG-CFI from both the perspectives of participants and clinicians. As a new mental disorder, PGD has stimulated clinicians and researchers to develop reliable and valid assessment measures. Currently there are new standardized questionnaires that are proving to be robust and valid assessments of PGD (Killikelly et al., 2020; Lenferink et al., 2022; Prigerson et al., 2021); however, a clinical interview is still lacking. Here we provide an in-depth exploration of clinicians’ and participants’ experiences with the first implementation of the semi-structured interview procedure of the BG-CFI.

Culture is a crucial component for understanding the expression of psychological distress and mental disorders. Cultural differences must be understood and considered for appropriate diagnosis and treatment (Bhugra & Becker, 2005). To bridge the possible cultural incongruency between clinician and patient, the latest diagnostic manuals have included well-structured interview guides and protocols, for example, the DSM-5 Cultural Formulation Interview (CFI). The BG-CFI was developed as a supplement to the CFI, which aims to examine cultural and contextual influences on mental health conditions. Jones-Lavallée et al. (2023) conducted a recent scoping review and found plentiful evidence for the feasibility, acceptability, and utility of the CFI across a variety of different settings and cultural contexts.

In terms of feasibility, the current findings on the BG-CFI mirror recent findings from the original CFI. The CFI was generally found to increase rapport and allow for expression of the patient’s illness experience (Jones-Lavallée et al., 2023). For example, at an outpatient clinic in Mexico, 19 patients were assessed with the CFI including debrief interviews for clinicians and patients. It was found that the CFI enhanced trust building between clinicians and patients and increased clinicians understanding of socio-cultural contextual influences on mental illness; however, the questions related to culture were deemed unhelpful or of limited value (Ramírez Stege & Yarris, 2017). In the current study, the BG-CFI was found to feasible with a few important recommendations. The interview was generally found it to be understandable, however, both participants and clinicians noted the questions elicited feelings of sadness, distress and strong emotions for several participants. The severity of distress was not deemed to be more than everyday life. However, when conducting the interview, clinicians and participants should be informed about the possibility of strong emotions and support should be offered. Clinicians also noted that care should be taken to avoid retraumatizing participants and to only conduct the interview with stable patients.

In terms of acceptability, generally the interview was found to be of tolerable duration, the content was found to be relevant, and none of the questions were deemed inappropriate with some suggestions for further clarifications of questions (e.g. questions 4, 7 on family and community grief reactions were deemed confusing). One important finding was participants’ experience that the interview structure permitted self-expression. This could be, for example, due to active listening of the clinician/researcher to the patient’s story or because of the patient’s possibility to speak openly about the topic. This goes in line with Singla et al. (2017) who found that techniques such as empathy, active listening, and collaboration were among the five most endorsed nonspecific elements used in effective psychological treatments.

In terms of clinical utility, the clinician perspective showed that the BG-CFI served as a helpful instrument to address culture-specific aspects of a patient’s experience. However, some difficulties were noted with translation, cultural differences, and complexity of interview questions. Similarly, Jones-Lavallee et al. (2023) also highlighted that Zhbidat et al. (2020) considered the core CFI questions to be too abstract and complicated. In terms of acceptability, Jones-Lavallée et al. (2023) generally found the CFI to be culturally sensitive, but Zhibat et al. (2020) modified the core CFI to screen for somatization, acknowledging this is a common phenomenon among refugees that have experienced trauma. In the current study with the BG-CFI, the participants also generally found the questions to be culturally sensitive. Suggestions were made to improve the overall comprehensive nature of the BG-CFI by asking about personal growth after grief and avoiding the presumption a grieving process has been initiated. In terms of utility, Jones-Lavallée et al. (2023) generally found that the CFI aids in diagnostic and treatment planning. In the current study, clinician feedback on the BG-CFI similarly suggested that the BG-CFI has clinical utility in exploring PGD, may assist participants in expressing their emotions and/or thoughts, and may help differentiate between PGD from PTSD.

Clinical utility of the BG-CFI not only relates to diagnostic utility, but also to its utility in treatment. Most recently, an additional study explored the question of how the use of the BG-CFI can contribute to culturally sensitive treatment of persistent and traumatic grief. A qualitative study among 8 therapists with experience offering psychotherapy for persistent and traumatic grief to culturally diverse patients was conducted (Smid et al, in press). The study yielded 6 themes: understanding grief in context, thinking in possibilities, adapting therapy, personal relationship with cultural traditions, avoidance versus culture, and shared decision-making. First, the BG-CFI promoted understanding grief in context, enabling therapists to assess what was appropriate within the patient’s culture and what not. Second, it helped therapists and patients to explore how therapy components that may be culturally inappropriate (e.g. talking or crying about the dead) could still be applied within the therapy setting. Third, it helped adapt therapy to accommodate cultural beliefs and norms, e.g. by integrating culturally appropriate rituals. Fourth, the BG-CFI allowed exploration of the patient’s personal relation to their cultural bereavement and grief traditions, creating space for helpful grieving behaviors that were less common within the patient’s culture. Fifth, it helped therapists to distinguish between patient avoidance and cultural norms, minimizing the risk of therapist avoidance while supporting culturally sensitive, grief-focused treatment. Sixth, the BG-CFI contributed to shared decision-making about grief treatment interventions based on shared insights in the cultural context, promoting the treatment alliance.

### Limitations

The small sample size of eleven bereaved participants and three clinician participants is a limitation of this study. The small sample size limits the generalizability in terms of feasibility, acceptability, and clinical utility across different cultures, languages and populations. Another limitation of the qualitative approach utilized is possible ambiguities in language and personal expression. For instance, a participant noted that the interview experience changed their perception of mental health care but not did not elaborate further. Language barriers and difficulties with translation may contribute to ambiguity in semantics. Additionally, since in the community stream of this study, the same researcher conducted the BG-CFI and the debriefing interview, some results may be affected by social desirability bias.

While only three clinicians offered their views in this study, their reflections were critical to our understanding of whether and how the tool could be used in practice. They raised key findings, which are important elements in understanding the complexity that clinicians navigate when diagnosing PGD and PTSD for bereaved patients. We therefore recommend further research to extend on clinician perspectives. Additionally, a diagnosis of PGD was not required to participate in this study. Follow up research should include participants with a confirmed clinical diagnosis of PGD to further validate the clinical utility of the BG-CFI.

### Implications

The BG-CFI recognizes the value of acknowledging social context and culture in the evaluation of PGD. Cultural ways of dealing with bereavement and grief are implicated in the attribution of meaning to a loss (Smid, 2020), e.g. performing appropriate rituals, religious/spiritual aspects, and the explanatory model regarding experiences related to grief. The potential impact of cultural factors on grief processes is supported by studies reporting cross-national differences in prevalence rates of PGD (Comtesse et al., 2024) and associations between ethnic minority status and the longitudinal course of mental distress following the loss of a loved one in disaster-affected residents (Smid et al., 2018a, 2018b). This pilot study of the BG-CFI suggests further changes and research will be needed to refine the interview. Suggested changes included: directing the questions about traditions (1-7) first more generally at how one handles a loss in one’s community and then specifically at the losses that the interviewed experienced; the addition of questions on previous grieving experiences, and about experiences of personal or spiritual growth since the death of a loved one.

Possible areas for improvement and future research could include evaluating diagnostic accuracy of the BG-CFI, the impact of BG-CFI on establishing participant-clinician rapport, and evaluating barriers to use in clinical practice. Nonetheless, our findings show promise for the feasibility, acceptability, and clinical utility of the BG-CFI as a cross-cultural assessment tool. The BG-CFI is developed as a standalone assessment tool that may be used independently of the larger CFI. This is to provide clinicians with a concise, easy to administer and shorter assessment when clarity about the role of grief and culture for treatment planning and assessment is required.

## Supplementary Information

Below is the link to the electronic supplementary material.

Supplementary file1 (DOCX 57 KB)

## Abbreviations

BG-CFI: Bereavement and Grief – Cultural Formulation Interview
CCD: Cultural concepts of distress
CFI: Cultural Formulation Interview
CPTSD: Complex Posttraumatic Stress Disorder
DSM-5-TR: Diagnostic and Statistical Manual of Mental Disorders (5th edition – text revision)
ICD-11: International Classification of Disorders (11th edition)
PGD: Prolonged Grief Disorder
