# Supplementary material for: Feasibility, Acceptability and Clinical Utility of the Bereavement and Grief Cultural Formulation Interview for Prolonged Grief Disorder
Source: Cult Med Psychiatry. 2025 Jul 28;49(4):1150–67. doi: 10.1007/s11013-025-09927-2 (PMC12745315; doi:10.1007/s11013-025-09927-2)
Supplement: Supplementary file 1 — Supplementary file1 (DOCX 57 KB) [file 11013_2025_9927_MOESM1_ESM.docx]

**Bereavement and Grief Cultural Formulation Interview (BG-CFI) (Smid et al., 2018)**

**GUIDE TO INTERVIEWER:** *The following questions aim to clarify key aspects of loss of loved ones, bereavement and grief from the point of view of the individual and other members of the individual’s social network (i.e., family, friends, or others). In order to identify grief-related problems, probe for (1) the loss of loved ones as a potential cause of the problem (2) loved ones being missing persons as potential additional stressor, especially following migration (3) rituals that could not be performed as a potential aspect of cultural identity causing concerns, difficulties, and feelings of guilt.*

*Make a distinction between natural vs traumatic loss and responses*

*A distinction should be made between reponses reflecting general cultural experiences and experiences of the individual*

*In conflict situations, make a distinction of loss before, during or after the conflict*

*Consider asking about specific gender differences*

*Consider the stability of the patient, some of the questions may be triggering in terms of reflecting on traumatic and grief responses. The patient should have a period of stabilization and grounding work prior to conducting the interview if possible. If the patient becomes distressed during the interview please use clinical judgement to withhold or rephrase questions.*

*This interview should be adapted for cases of ambiguous loss*

**INTRODUCTION FOR THE INDIVIDUAL BEING INTERVIEWED**: Sometimes peoples’ background affects how they deal with the loss of loved ones. In order to better help you, I would like to learn to understand the way you, your family, friends, and others in your community deal with the loss of loved ones.

Interview Questions

**How has the grieving process been for you so far?**

**Cultural traditions related to death, bereavement, and mourning**

1. If someone from your family, friends, or others in your community passes away, how would people usually arrange the funeral/farewell?

   (PROBE AS NEEDED: Is there a prescribed period after death that a person should be buried or cremated?)
2. Are there other rituals after people have passed away, for example, do people hold a wake? Do people find it important to perform these rituals? And if yes, why?
3. Who should be present at these rituals and/or the funeral/farewell and why is that important?
4. How do your family, friends, and others in your community mourn or express their grief after the funeral/farewell? For example what do people do and how do they cope?

   Could you tell me about a prescribed period of mourning or expressing grief?

   When would it be considered a problem?
5. When and how do people talk about the deceased?

   (PROBE AS NEEDED: Is it considered appropriate to talk about the deceased?)

   Are there certain moments when the deceased is remembered, such as yearly commemorations? Can you tell me more about this?
6. When bereaved people have dreams or other types of encounters with the deceased (e.g., seeing the deceased or talking to him or her), what could that mean?

   And what would this mean according to your family, friends, and others in your community?
7. What do your family, friends, and others in your community think happens after death?

   (PROBE AS NEEDED: Does it matter in what way the loved one died, e.g. in case of suicide? Could you elaborate on that?)

   Are there ways in which the living can influence the spiritual status of the deceased in the afterlife?

Are there ways in which the deceased can influence the living? Can you tell me more about this?

**Help-seeking and coping related to the loss of loved ones**

1. Do you engage in practices or take part in activities related to particular spiritual, religious or moral traditions to help you cope with the loss of a loved one (e.g., prayer, meditation, worships, gatherings, or talking to a religious or spiritual leader)?
2. Have any of these practices or activities been helpful in coping with the loss of your loved one (e.g., in dealing with guilt feelings or anger)? In what way?
3. Are there other kinds of help to deal with the loss of your loved one that your family, friends, or other people have suggested?

   What kinds of help do you think would be most useful to you at this time to deal with the loss of your loved one?

**Additional questions***

1. Do you grieve differently at home, compared to when in host country (Switzerland)? How, why?
2. Is there anything else that you think we should know?

Have you noticed any positive developments in yourself since the loss? How have you changed since the loss in terms of resilience, strength or recovery?

For original and translated versions of the BG-CFI, please visit:

Smid, G. E., Killikelly, C., & Groen, S. (2024, June 25). Bereavement and Grief—Cultural Formulation Interview (BG-CFI). Retrieved from osf.io/w7svh

© Smid, G. E., Groen, S., de la Rie, S. M., Kooper, S., & Boelen, P. A. (2018). Toward cultural assessment of grief and grief-related psychopathology. Psychiatric Services, 69(10), 1050–1052. <https://doi.org/10.1176/appi.ps.201700422> [online supplement]

*© Additional questions: C. Killikelly et al. 2019

C. Killikelly et al., 2025 Revisions following recommendations from clinicians and bereaved participants as published in **Feasibility, acceptability and clinical utility of the Bereavement and Grief Cultural Formulation Interview for Prolonged Grief Disorder, Culture Medicine and Psychiatry**
